# Emergence of symmetry selectivity in the visual areas of the human brain: fMRI responses to symmetry presented in both frontoparallel and slanted planes

**DOI:** 10.1002/hbm.24211

**Published:** 2018-07-03

**Authors:** Bruce D. Keefe, André D. Gouws, Aislin A. Sheldon, Richard J. W. Vernon, Samuel J. D. Lawrence, Declan J. McKeefry, Alex R. Wade, Antony B. Morland

**Affiliations:** ^1^ York Neuroimaging Centre, Department of Psychology University of York York United Kingdom; ^2^ School of Optometry & Vision Sciences University of Bradford Bradford United Kingdom; ^3^ Centre for Neuroscience, Hull‐York Medical School York United Kingdom

**Keywords:** fMRI, occipital lobe, retinotopy, symmetry, visual areas, visual cortex

## Abstract

Symmetry is effortlessly perceived by humans across changes in viewing geometry. Here, we re‐examined the network subserving symmetry processing in the context of up‐to‐date retinotopic definitions of visual areas. Responses in object selective cortex, as defined by functional localizers, were also examined. We further examined responses to both frontoparallel and slanted symmetry while manipulating attention both toward and away from symmetry. Symmetry‐specific responses first emerge in V3 and continue across all downstream areas examined. Of the retinotopic areas, ventral occipital VO1 showed the strongest symmetry response, which was similar in magnitude to the responses observed in object selective cortex. Neural responses were found to increase with both the coherence and folds of symmetry. Compared to passive viewing, drawing attention to symmetry generally increased neural responses and the correspondence of these neural responses with psychophysical performance. Examining symmetry on the slanted plane found responses to again emerge in V3, continue through downstream visual cortex, and be strongest in VO1 and LOB. Both slanted and frontoparallel symmetry evoked similar activity when participants performed a symmetry‐related task. However, when a symmetry‐unrelated task was performed, fMRI responses to slanted symmetry were reduced relative to their frontoparallel counterparts. These task‐related changes provide a neural signature that suggests slant has to be computed ahead of symmetry being appropriately extracted, known as the “normalization” account of symmetry processing. Specifically, our results suggest that normalization occurs naturally when attention is directed toward symmetry and orientation, but becomes interrupted when attention is directed away from these features.

## 1 INTRODUCTION

A visual system exquisitely tuned to symmetry is essential if we are to successfully interact with a biological environment in which it is abundant. Because asymmetry in one's conspecifics can indicate acquired or congenital disease, symmetry processing also plays a unique role in mate selection and survival. Owing to these strong evolutionary pressures, the human visual system has evolved a network of symmetry sensitive areas, emerging as early as V3 and extending across extrastriate visual cortex (Bertamini & Makin, 2014; Bona, Herbert, Toneatto, Silvanto, & Cattaneo, 2014; Chen, Kao, & Tyler, 2007; Kohler et al., 2016; Makin, Rampone, & Bertamini, 2015; Sasaki et al., 2005; Tyler et al., 2005). Recent years have seen new retinotopic areas discovered and greater consensus reached on the canonical layout of visual cortex (Amano, Wandell, & Dumoulin, 2009; Brewer, Liu, Wade, & Wandell, 2005; Larsson & Heeger, 2006; Wandell, Brewer, & Dougherty, 2005; Winawer et al., 2010). It is timely therefore to re‐examine neural responses to symmetry based on our current understanding of retinotopic visual cortex. To this end, utilizing frontoparallel stimuli, we varied both symmetry coherence and folds of symmetry while examining responses across retinotopically defined V1, V2, V3, V3AB, V7, LO1, LO2, TO1, TO2, V4, VO1, and VO2 and functionally defined lateral occipital complex (LOC), split into dorsal/posterior LOB and ventral/anterior pFs. To re‐examine the role of attention, we chose a task manipulation different to previous symmetry experiments (Sasaki, Vanduffel, Knutsen, Tyler, & Tootell, 2005), by borrowing from recent experiments that have examined changes in viewing geometry (Makin et al., 2015). Specifically, we compared responses during passive viewing to responses when a symmetry detection task was performed.

While studying symmetry in the frontoparallel plane affords the control of the perfect retinal symmetry it projects, it offers little ecological validity. Rarely do we experience symmetry confined to this canonical orientation in our natural world. Rather, we experience a world awash with symmetry across surfaces slanted in depth. A symmetry detector only sensitive to perfect retinal symmetry would thus fare poorly, and could not produce the perception of symmetry that we each experience. Specifically, bilateral symmetry in the frontoparallel plane contains both first‐ and second‐order structures that cast retinal regularities. First‐order structure comes from corresponding points that produce virtual parallel lines with collinear midpoints (Jenkins, 1983), and second‐order structure comes from pairs of virtual lines that form symmetrical trapezoids (Wagemans, van Gool, & D'ydewalle, 1991; Wagemans, Van Gool, Swinnen, & Van Horebeek, 1993). While these retinal first‐ and second‐order regularities likely aid detection of symmetry in the frontoparallel plane, both are significantly degraded by perspective shifts around the axis of symmetry (Wagemans, 1993). Inspired by theoretical work originally detailing the importance of these regularities, psychophysical and electrophysiological studies have started to examine the effects of perspective shifts on symmetry perception. Symmetry detection is found to deteriorate, both when symmetry is shifted away from the frontoparallel plane (Locher & Smets, 1992; van der Vloed, Csathó, & van der Helm, 2005) and when binocular depth cues are removed from symmetry slanted in depth (Szlyk, Rock, & Fisher, 1995). Moreover, reaction times during symmetry detection have been shown to increase as a function of perspective shifts away from the frontoparallel plane (van der Vloed et al., 2005). Because perspective both distorts retinal regularities and hinders behavioral performance, it has been suggested that the visual system may perform a normalization process under slanted viewing conditions. Specifically, this process would require that the visual system first obtain an estimate of slant from available depth cues to mentally rotate (normalize) the image to the frontoparallel plane where regularity detection could proceed unhindered (on this mentally rotated image, rather than the retinal image). Others however, have suggested that during perspective distortions, the remaining regularities in the retinal image are sufficient for symmetry detection to take place, without requiring normalization, a process typically referred to as the retinal structure account of symmetry processing (Jenkins, 1983; van der Vloed et al., 2005; Wagemans et al., 1993). Attempts to discern between these two putative mechanisms have returned equivocal conclusions in the psychophysical literature however, and we refer the interested reader to the study by van der Vloed et al. (2005) for an excellent discussion on the topic.

Interestingly, the normalization and retinal structure accounts generate straightforward predictions for neural studies looking to test between them. Normalization, being an effortful computation that draws on neural resources may only be performed when strictly necessary, such as when one attends to slanted symmetry. Under conditions in which attention is directed off stimulus, however, normalization may become interrupted as estimates of slant degrade and activity is driven primarily by regularities in the retinal image. These regularities, being distorted by perspective, would produce weaker signals than during active normalization. Strict retinal regularity accounts of symmetry processing however do not predict that the pattern of responses to symmetry in different planes should change when different tasks are employed. To test these predictions, Makin et al. (2015) recorded symmetry‐related ERPs while participants viewed symmetry on the frontoparallel and slanted planes. Attention was directed either toward or away from the symmetry in the stimulus using regularity discrimination and color discrimination tasks, respectively. Similar ERPs would be expected if normalization takes place but not if normalization becomes interrupted owing to task demands. Indeed, the ERP component was found to be viewpoint invariant when attending to the symmetry in the stimulus, but to reduce for slanted presentations when participants discriminated color. Under the latter conditions it is likely that normalization became interrupted and the symmetry network responded primarily to the preserved retinal regularity in the image, which degraded by perspective, resulted in lower ERP responses.

After Makin et al. (2015), we performed experiments in which we presented participants with onefold symmetrical patterns in both the frontoparallel and slanted planes (± 50°). In one experiment, participants performed a symmetry detection task, while in the other, they performed a color detection task. We examined responses in the same subjects and thus the same retinotopic and functionally defined areas as in the experiments conducted solely on the frontoparallel plane. The same visual areas were found to be active for slanted and frontoparallel symmetry and like Makin et al. (2015), we found similar responses to these two orientations for a symmetry‐related task. The color task, however, was found to compromise responses to slanted symmetry, suggesting that normalization is interrupted when attention is directed away from symmetry and orientation.

## 2 MATERIALS AND METHODS

### 2.1 Participants

Eight participants were recruited to the study of whom seven (4 female, aged 22–38) completed retinotopic mapping sessions, functional localizers, psychophysics, and the symmetry fMRI experiments reported here. All participants had normal or corrected‐to‐normal visual acuity and no history of neurological impairments. Informed consent was obtained in accordance with the Declaration of Helsinki. Procedures and protocols were approved by the York Neuroimaging Centre (YNiC) Research Ethics Committee at The University of York, United Kingdom. Each participant underwent 8 h of MRI scanning (as described below) to examine our questions.

### 2.2 Retinotopic Mapping Procedures

**Figure 1:**
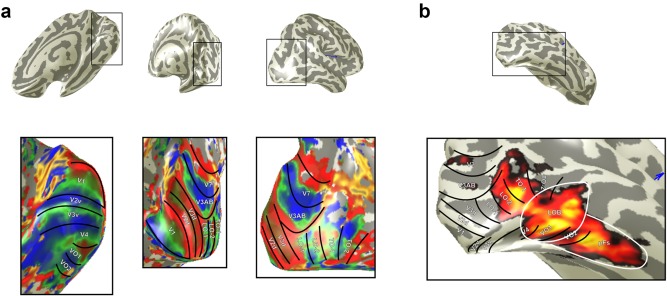
(a) Retinotopically defined ROIs. Upper row shows the inflated right hemisphere for one participant. Bottom row shows the same inflated hemisphere with overlaid color map detailing polar angle data from rotating wedge stimuli. Visual areas are subsequently drawn and labeled according to the reversals in phase that demarcate them. (b) Heat map from the localizer experiment. LOB and pFs are drawn in white

Crucial to our fMRI experiments was the need to first identify retinotopic maps in each individual. For the purposes of this study, we set out to identify V1, V2, V3, V3AB, V7, LO1, LO2, TO1, TO2, V4, VO1, and VO2 in each hemisphere (Figure 1). We used two established methods to obtain retinotopic data. For four of our participants, a phase encoded approach was employed in which a rotating wedge was used to map polar angle, expanding rings were used to map eccentricity, and standard Fourier methods were used to analyses the retinotopic data (DeYoe et al., 1996; Engel, Glover, & Wandell, 1997; Tootell et al., 1997). For the remaining three participants, we used population receptive field (pRF) mapping (Dumoulin & Wandell, 2008), in which both rotating wedge and drifting bar stimuli with blank periods contributed to the final model fit. For the phase‐encoded approach, stimuli were unmasked portions of a 100% contrast radial dartboard pattern checkerboard (14° radius) with 24 radial segments on a mid‐grey background. Wedges were 90° in size and rotated counterclockwise about a red fixation cross; ring stimuli expanded about fixation. Stimuli reversed contrast at a rate of 6 Hz and participants maintained fixation throughout the scan. Eight scans were collected (4 wedges, 4 rings; counterbalanced) and each scan contained 8 cycles of wedges/rings, with 36 s per cycle. For the pRF approach, wedge stimuli were unmasked portions of a 100% contrast radial dartboard pattern checkerboard (11° radius) with 24 radial segments on a mid‐grey background. Wedges were 45° in size and rotated counterclockwise about a red fixation circle. Each scan contained 6 cycles of wedges, with 63 s per cycle. Bar stimuli were masked portions of a 100% contrast square checkerboard (11° radius). The bar width subtended one‐fourth of the stimulus radius (2.75°) and transversed the visual field through 4 cardinal directions (horizontal, vertical, and diagonals) and two motion directions, providing 8 complete movements per scan. Each of these 8 movements comprised 16 movements steps, each lasting 3 s and updating on the TR such that each complete bar movements lasted 48 s. For both wedges and bars, mean‐luminance periods were included in which the observer saw only a mean‐luminance (mid‐grey) screen at 4 cycles/scan. A total of eight scans were collected (6 bars, 2 wedges; counterbalanced). All stimuli used were high contrast (>98%, 400 cdm^−2^) checkerboard stimuli. For the phase encoded approach data were averaged across scan type (ring/wedge) and for the pRF approach, data were averaged across scan type (wedge/bar), a single pRF model was then fit to these averages.

Gradient‐recalled echo pulse sequences were used to measure BOLD signals acquired parallel to the calcarine sulcus. For the retinotopy data (TR = 3,000 ms, TE = 30 ms, flip angle = 90°, FOV = 192 × 192 × 78, 96 × 96 matrix, 39 contiguous slices per volume at 2 × 2 × 2 mm). The first three volumes from all scans were discarded to allow the magnetization to reach a steady state.

Functional data across all sessions were aligned to a canonical anatomical volume using a proton‐density image acquired with the same prescription as the functional data as an intermediate alignment step. Motion correction was achieved using FSL's MCFLIRT (Jenkinson, Bannister, Brady, & Smith, 2002) and no significant movements were seen throughout scanning. The functional time series were high‐pass filtered to remove baseline drifts. We used mrVista and mrMesh analysis software to perform the retinotopic analysis and visualize data in volume and inflated cortical views (http://white.stanford.edu). Visual areas were hand drawn on these inflated cortical views according to established reversals in polar angle demarcating specific visual areas (Amano et al., 2009; Brewer et al., 2005; Larsson & Heeger, 2006; Wandell et al., 2005; Winawer et al., 2010; Figure 1).

For the anatomical data that provided a canonical volume, we used a procedure to increase tissue contrast for automated segmentation: Three whole‐head T1‐weighted anatomical volumes were acquired for each subject (TR = 7.8 ms, TE = 2.7 ms, TI = 600 ms, flip angle = 12°, FOV = 256 × 256 × 176, 256 × 256 × 176 matrix, 1 × 1 × 1 mm^3^) using a 16‐channel (half‐head coil) and averaged. One T2*‐weighted fast gradient recalled echo scan was also acquired (TR = 400 ms, TE = 4.3 ms, flip angle = 25°, field of view = 290 × 290 × 176, 256 × 256 × 88 matrix, 1.13 × 1.13 × 2 mm^3^) using a 16‐channel head coil. Average T1 data were divided by the T2* data to correct for signal gradient resulting from the signal dropout of the 16‐channel coil and to improve white/gray matter contrast. One whole‐head 8‐channel T1‐weighted volume was acquired for each subject (TR = 7.8 ms, TE = 2.9 ms, TI = 450 ms, flip angle = 20°, FOV = 290 × 290 × 176, 256 × 256 × 176 matrix, 1.13 × 1.1.13 × 1 mm^3^). The average T1‐weighted anatomical volume was segmented into white and gray matter for each hemisphere using Freesurfer (http://surfer.nmr.mgh.harvard.edu/). The subsequent gray–white matter segmentation was hand edited and checked for topology errors using itkGray (http://white.stanford.edu).

### 2.3 Functional Localizer

We performed experiments to identify object selective cortex, conventionally referred to as the lateral occipital complex (LOC) (Grill‐Spector, Kourtzi, & Kanwisher, 2001; Malach et al., 1995). The LOC is a relatively large swathe of cortex that has a posterior and more dorsal portion, which has been referred to as LO and a more ventral and anterior portion that is referred to as posterior fusiform gyrus (pFs; Grill‐Spector et al., 1999). A further subdivision of LO has been based on two relatively distinct regions one lying most dorsal and posterior (LOA) and the other more ventral and anterior (LOB) and lying between LOA and pFs (Vinberg and Grill‐Spector, 2008). It is further noted that previous work has shown considerable overlap between LO and the retinotopic areas LO1 and, more particularly, LO2 (Larsson & Heeger, 2006; Sayres, & Grill‐Spector, 2008). We selected the regions LOB and pFs as regions of interest in addition to the regions that we identified with retinotopic mapping for two reasons: (1) Only these areas were reliably identified in all participants and (2) in the six participants in whom we were able to identify LOA, we found it overlapped with LO2. Overlap of LOB and pFs with our retinotopic areas was considerably less, although where overlap was found it was mainly between VO1 and LOB. A particularly striking example of such overlap is shown for an individual participant's ROIs (Figure 1).

For the functional localizer, three 8 min localizer scans were employed (using identical imaging parameters to those used in the retinotopic scans; see above). An ABAB block design which contrasted objects with scrambled objects was used. Each scan comprised 16 object blocks and 16 scrambled object blocks (15 s blocks), with one image presented per second (0.8 s presentation, 0.2 s interstimulus interval). Participants maintained fixation on a central red cross while performing a one‐back task in which there could be one, two, or no repeats within a given block (to ensure that attention was maintained). All stimuli were presented centrally on a full‐screen mid‐gray background (200 cdm^−2^), and there were no baseline/rest periods between blocks. Stimuli comprised 225 PNG images of easily recognizable objects, manually extracted from their original backgrounds. These were converted to grayscale with a flattened (equalized) image histogram. On average stimuli subtended 4 × 4° of visual angle (exact size depended on image aspect ratio). To create scrambled stimuli, we split the objects and background into a grid with 20 rows and columns (square size 0.8° × 0.8°), and then all squares lying within the convex hull of the object were randomly permutated and rotated. This meant scrambled objects would contain all local details from the original objects, plus the same coarse outline, but would not be semantically recognizable or symmetrical. Because scrambling introduced sharp contrast edges between permuted squares, we applied a Gaussian filter (SD of 1 pixel) to both the objects and scrambled objects.

Localizer data were analyzed using FEAT. At the first (individual) level, we removed the first three volumes and used a high‐pass filter cutoff point of 60 s to correct for low‐frequency drift. Spatial smoothing was performed with a Gaussian kernel of 4 mm FWHM, and FILM prewhitening was used. To combine data within a participant, we ran fixed‐effects analysis with cluster correction (*Z* > 2.3, *p* > .05). LOB and pFs were subsequently defined based on the resulting activation (Figure 1).

### 2.4 Symmetry stimuli and procedure

#### 2.4.1 Stimuli to explore symmetry coherence and folds of symmetry on frontoparallel planes

**Figure 2:**
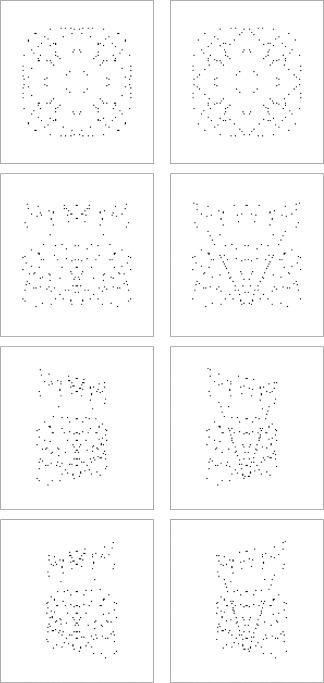
Example stimuli are shown with and without the embedded target motif used in the symmetry experiments. The left column shows stimuli without the target and the right column show the same stimuli with the target embedded. The first row shows fourfold, frontoparallel stimuli, with and without an embedded fourfold diamond motif. The second, third, and fourth row show onefold stimuli, with and without an embedded onefold V‐target motif, in the frontoparallel, positively slanted (+50°), and negatively slanted (−50°) planes, respectively

**Figure 3:**
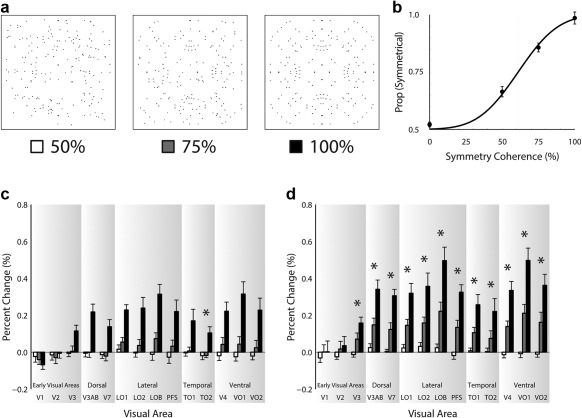
Panel (a) shows exemplar 4‐fold stimuli for 50%, 75%, and 100% coherence and panel (b) shows group psychophysical performance to these stimuli. Panels (c) and (d) show the fMRI results for the passive viewing and symmetry detection experiments, respectively. Visual areas are labeled on the abscissa and percent signal change is shown on the ordinate. Responses to the 50% coherence stimuli are represented by white bars, 75% coherence stimuli by grey bars, and 100% coherence stimuli by black bars. Error bars show ± SEM. Asterisks indicate significant linear trend

**Figure 4:**
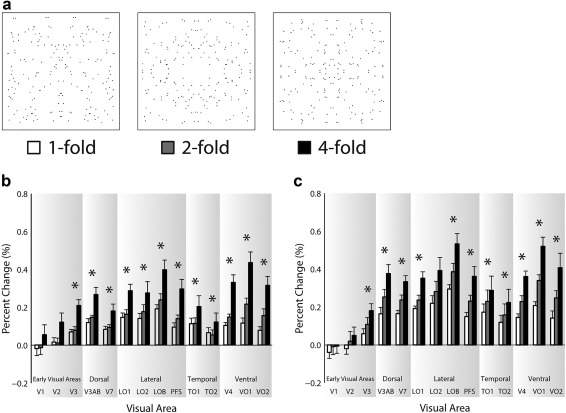
Panel (a) shows exemplar stimuli for one‐, two‐, and fourfold 100% coherence symmetry. Panels (b) and (c) show the fMRI results for the passive viewing and symmetry detection experiments, respectively. Visual areas are labeled on the abscissa and percent signal change is shown on the ordinate. Responses to the onefold stimuli are represented by white bars, twofold stimuli by grey bars, and fourfold stimuli by black bars. Error bars show ± SEM. Asterisks indicate a significant linear trend

Sparse symmetrical patterns (1.8% density as defined by the percentage of red dot pixels in the entire stimulus, giving 0.7 dots per deg^2^) were generated by distributing red dots on a mid‐grey background after Sasaki et al. (2005). Here we restricted the dots to a single‐color channel (red) to reduce chromatic aberrations at the projector screen and render high fidelity stimuli. Stimuli were a square, 16° on a side and each dot was 0.16° wide. We generated stimuli to examine symmetry coherence, folds of symmetry, symmetry from slanted planes, and the contribution of attention to symmetry processing. To examine symmetry coherence we chose a fourfold stimulus to maximize the neural response. Four conditions were used: 100% symmetry; 75% symmetry; 50% symmetry, and 0% symmetry (100% noise) (Figure 3). Dot patterns were first generated with 100% symmetry, a proportion of dots corresponding to the appropriate noise level were then removed and randomly replaced at previously unoccupied locations across the stimulus. To probe folds of symmetry, four conditions were used: fourfold stimuli containing horizontal, vertical, and diagonal symmetry; twofold stimuli containing horizontal and vertical symmetry; onefold stimuli containing vertical symmetry, and 100% noise stimuli containing no symmetry (Figure 4). In separate experiments, the role of attention was probed by replacing passive viewing with a symmetry detection task. During these sessions, catch trials were included in which a motif was embedded within the stimulus and participants were asked to count the total number of motifs presented within each scan. We used a counting task for simplicity and to avoid potential confounds introduced by button presses. For fourfold and twofold patterns, a diamond motif whose points touched the center, top, bottom, and sides of the stimulus was embedded (Figure 2). For onefold patterns, a V‐shape that ran from the top edges to the bottom center of the stimulus was embedded (Figure 2). Importantly, by choosing motifs that were themselves symmetrical, we ensured that attention was maintained on a symmetry relevant, rather than symmetry irrelevant feature. To make the motif stimuli, we first positioned the dots constituting the symmetry stimulus (80% of total dots), while masking the area in which the motif could fall. The remaining 20% of dots were then randomly distributed along the motif line in a fashion that preserved the folds of symmetry present in the stimulus (Figure 2).

#### 2.4.2 Stimuli to compare frontoparallel and slanted symmetry

To explore slanted symmetry, we used the same 100% coherence, onefold, frontoparallel stimulus used to study symmetry folds and included slanted versions where 50° of positive or negative slant was applied about the symmetry axis of the stimulus (also known as a Y‐rotation; Figure 2). Onefold stimuli were chosen because they eliminate the perfect retinal symmetry found along the horizontal axis of two‐ and fourfold stimuli. To help understand this, we can imagine cutting out the stimuli in Figure 4 and folding along their horizontal axis. Both two‐ and fourfold stimuli fold to match their upper and lower halves, whereas onefold stimuli do not. By using onefold slanted symmetry, we therefore purposely restrict our investigation to symmetry slanted in depth (rather than a combination of symmetry present in both the frontoparallel and slanted planes as is the case for slanted two‐ and fourfold stimuli). The role of attention was again probed by having participants perform a symmetry‐related task using the same V‐motif as described earlier (Figure 2). In another experiment, instead of including the V‐motif, the luminance of the stimulus was manipulated on the same catch trials using a luminance decrement on the red channel from 255 to 215; the mid‐grey background was unchanged. These separate experiments were completed on separate days. As in the previously described experiments, participants were asked to count the number of motifs, or luminance decrements, and verbally report this count at the end of each block.

#### 2.4.3 Procedure for experiments examining symmetry coherence and folds of symmetry on frontoparallel planes

Stimuli were generated using Matlab and Psychtoolbox (Brainard, 1997; Kleiner et al., 2007) and were rear projected onto an acrylic screen and viewed by participants lying supine in the scanner from 57 cm via a front‐silvered mirror mounted onto the MRI head coil. During all experimental scans reported here, a small black fixation cross (0.2°) was present at the center of the stimulus which participants were instructed to fixate. Both symmetry coherence and symmetry folds were examined in the same scanning session. Each session began with a stimulus localizer scan, followed by six experimental scans (three for each of coherence and folds). The experimental scans were blocked by symmetry type (coherence or folds) and counterbalanced across participants such that the first three scans in a session were either all coherence or all folds scans. To allow us to probe the role of attention, each participant undertook two scanning sessions, completed on separate days. In one of these sessions the participant was required to count the number of symmetry‐related targets and report these at the end of the block (described earlier and shown in Figure 2). The other session required participants only to passively view the stimulus. The order of these two scanning sessions was counterbalanced across participants.

Each experimental scan comprised of fifty 8 s blocks during which one of 5 conditions was presented. These 5 conditions included the 4 stimulus types (3 levels of stimulus plus noise; Figure 4), and a blank period. During stimulus presentation, the stimulus was updated every second providing 8 unique stimuli per block; for the blank period the screen remained mid‐grey. Each of these 5 conditions was repeated 10 times during a scan, with the order of conditions pseudorandomized, so that no two conditions were presented back‐to‐back. We repeated the scan session 3 times, obtaining 30 repetitions for each condition. Each scan session lasted 6 m 46 s, including 6 s of dummy volumes.

A stimulus localizer scan used to identify the retinotopic extent of the stimulus employed a 100% contrast radial checkerboard (8° radius) with 24 radial segments on a mid‐grey background. The checkerboard stimuli contrast reversed at 5 Hz. During the on period the radial checkerboard was restricted to the same 16° square commensurate with the stimulus location. During the off period, this central 16° square remained mid‐grey and the remainder of the checkerboard was visible. The on and off period were presented back‐to‐back lasting 8 s each. Each on/off period was repeated 10 times and the total scan duration was 166 s including 6 s of dummy volumes. It is noted here that this procedure was unsuccessful in identifying the stimulus representation in higher order visual areas (beyond V3 dorsally and V4 ventrally), where receptive fields sizes are larger (Amano et al., 2009; Dumoulin & Wandell, 2008). The data we present in the Results were obtained using a uniform approach that did not attempt to constrain signals to the stimulus representation, allowing for unbiased measures of symmetry selectivity across the visual cortex. To check if responses differed between analyses performed on the stimulus localizer restricted data and the unrestricted data, we compared these responses for early visual areas where stimulus localizer data were consistently obtained.

#### 2.4.4 Procedure for experiments comparing frontoparallel and slanted symmetry

**Figure 5:**
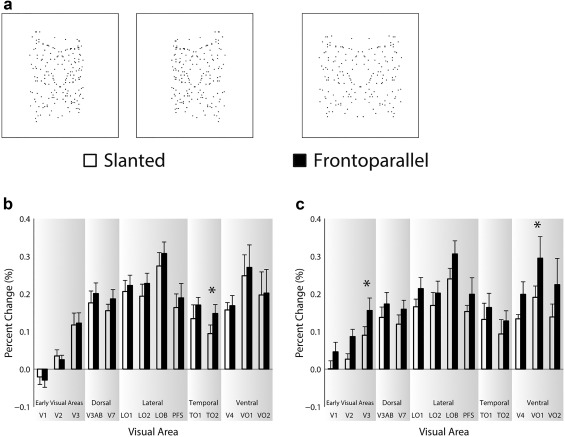
Panel (a) shows exemplar positively slanted (+50°), negatively slanted (−50°), and frontoparallel (0°) onefold stimuli. Panels (b) and (c) show the fMRI results for the symmetry and color detection tasks, respectively. Visual areas are labeled on the abscissa and percent signal change is shown on the ordinate. Error bars show ± SEM. Asterisks indicate a significant *t* test

For the experiments comparing frontoparallel and slanted symmetry, each scan session comprised of forty‐two 8 s blocks during which one of 7 conditions was presented. These 7 conditions included the blank period and the 6 stimulus types, namely, the symmetry stimuli for frontoparallel, positively, and negatively slanted planes (Figure 5), and the noise stimuli matched to the respective orientations of these planes. During presentation, the stimulus was updated every second providing 8 unique stimuli per block, while for the blank period, the screen remained mid‐grey for 8 s. Each of these 7 conditions was repeated 6 times during a scan, with the order of conditions pseudorandomized, so that no two conditions were presented back‐to‐back. We repeated the scan session 5 times, yielding 30 repetitions for each condition. Each scan sessions lasted 5 m 42 s, including 6 s dummy volumes.

To allow us to probe the role of attention, each participant undertook two scanning sessions completed on separate days. In one of these sessions, the participant was required to count the number of symmetry‐related targets (described earlier and shown in Figure 2) and report these at the end of the block. The other session required participants to count the number of luminance decrements. The order of these two scanning sessions was counterbalanced across participants.

#### 2.4.5 Stimulus localizer

We did not use a localizer scan for experiments comparing frontoparallel and slanted planes for two reasons. First, using a localizer would have been problematic owing to the variable retinotopic coverage between frontoparallel and slanted presentations. Second, as we discuss in the results, for the coherence and folds experiments utilizing frontoparallel stimuli, we found a strong, significant correlation between localizer‐restricted and unrestricted full‐field data for lower visual areas. This suggests that the pattern of results would be similar whether or not we chose to use a localizer to restrict analysis to the retinotopic locus of the stimulus. We are confident, therefore, to interpret the results for this set of experiments without using localizer‐restricted data.

#### 2.4.6 Motif trials

Motif trials in which symmetrical motifs were embedded in the stimulus were only presented within symmetry stimuli as to present them within noise stimuli would have added symmetry to the noise baseline. In the coherence and folds experiments, utilizing just frontoparallel stimuli, the motif target trials made up 4.7% of all trials containing dots (symmetry and noise stimuli), a trial being a single, one second presentation of the stimulus. The motifs were equally distributed across the three levels of the stimulus (100%, 75%, and 50% coherence; one‐, two‐, and fourfold). In the experiments comparing frontoparallel and slanted stimuli, the target trials made up 4.3% of all trials.

### 2.5 fMRI data collection and analysis

All imaging data involved in the process of retinotopic mapping and functional sessions were acquired on a GE 3‐Tesla Signa HD Excite scanner at the York Neuroimaging Centre, University of York. A 16‐channel head coil was used to improve signal‐to‐noise in the occipital lobe.

All functional data for symmetry were analyzed using FEAT (FMRI Expert Analysis Tool; Worsley, 2001). At the first level, we removed the first three dummy volumes and used a high‐pass filter cutoff point of 32 s to correct for low‐frequency drift. Spatial smoothing was performed with a Gaussian kernel of 6 mm FWHM, and FILM prewhitening was used. Functional volumes were motion corrected using MCFLIRT and aligned to individuals structural images via a proton density image acquired in the same session as the functional volumes. Contrasts were set up to compare each separate stimulus to its respective noise baseline (frontoparallel, or slanted). To combine data within a participant, we ran fixed effects analysis with cluster correction (*Z* > 2.3, *p* < .05). Percentage signal change was then computed by visual area using FeatQuery. The resulting data were then averaged across hemispheres for all ROIs and across ventral and dorsal V2 and V3.

Gradient recalled echo pulse sequences were used to measure BOLD signals acquired parallel to the calcarine sulcus. For the symmetry experiments, TR = 2,000 ms, TE = 30 ms, flip angle = 80°, FOV = 192 × 192 × 114, 64 × 64 matrix, 38 contiguous slices per volume at 3 × 3 × 3 mm. The first three volumes from all scans were discarded to allow the magnetization to reach magnetization steady state.

### 2.6 Psychophysics

#### 2.6.1 Procedure for psychophysics

To measure participants symmetry coherence thresholds, we performed a psychophysical experiment in which we used the same fourfold symmetry stimuli used in the fMRI experiment. Participants fixated a central black fixation cross (0.2°) throughout the experiment. On each trial, two test stimuli (a standard and comparison) were presented sequentially for 1 s each, with a 500 ms interstimulus interval. The screen then turned mid‐grey and participants indicated which of the two stimuli was more symmetrical using a key press. Following the participant's response, the screen remained blank for 500 ms before the next trial began. The standard stimulus was always a 0% coherence noise stimulus, and the comparison stimulus was a 100% coherence, 75% coherence, 50% coherence, or 0% coherence stimulus. Each level of the comparison stimulus was repeated 30 times within an experimental block and the trial order was pseudorandomized such that the same comparison coherence level was never repeated across successive trials. The order of the standard and comparison within each trial was randomized. Participants completed two experimental blocks, yielding a total of 60 repeats for each coherence level of the comparison stimulus. The experiment took ∼20 min to complete, split across two 10‐min blocks. A cumulative psychometric function was fitted to the pooled data from the seven participants and the error bars calculated to show the standard error for each coherence level across participants. Stimuli for the psychophysics were generated using Matlab and Psychtoolbox (Brainard, 1997; Kleiner et al., 2007) and displayed on a Mitsubishi Diamond Pro 2070^SB^ display (viewing distance = 57 cm) with a resolution of 1,600 × 1,200 pixels and a refresh rate of 85 Hz.

## 3 RESULTS

### 3.1 Experiments examining symmetry coherence and symmetry folds on frontoparallel planes

We show behavioral performance and brain responses to different levels of coherence of symmetry across the divisions of visual cortex in Figure 3. Coherence has a clear effect on psychophysical performance in detecting symmetry, being close to chance at 0% coherence and rising to ceiling performance at 100% coherence (Figure 3b). When participants passively viewed stimuli, symmetry sensitive responses were found throughout most visual areas for 100% coherence stimuli (Figure 3c). These responses first emerged in V3 and were consistently found in all downstream areas examined. Interestingly, it appears that stimuli with lower coherence elicited little or no response across visual cortex. When participants monitored the symmetrical motifs that rarely appeared in our stimuli, the response patterns differed in the following ways: (1) we now detected response for stimuli at 75% coherence and (2) responses were enhanced overall (Figure 3d). Responses during symmetry detection thus showed greater correspondence to the behavioral psychophysical results when participants actively discriminated symmetry and the just noticeable difference fell between the 50% and 75% coherence levels at 61%. However, as was observed for passive viewing, symmetry‐sensitive responses were found to begin in V3 and continue through all downstream areas examined. Across both passive viewing and symmetry detection experiments, we found the strongest symmetry responses in ventral occipital VO1 and in object selective cortex, LOB.

We were keen to examine our data statistically to test whether there were specific effects of our stimulus manipulations and tasks, and visual area, on the responses we recorded. We therefore performed three‐way ANOVAs for each experiment reported here. Our predictions are simple. If symmetry has an extrastriate origin, then we would expect to see symmetry‐specific responses in later, but not early visual areas, as evidenced by a main effect of visual area. If our stimulus manipulations (coherence, folds, and slant) differentially modulate symmetry responses, we could see a main effect of stimulus, but this is likely to be specific to those visual areas that exhibit symmetry response, which would emerge as an interaction term with visual area. Similarly, if the task deployed affects symmetry responses, then a main effect of task may be observed, but an interaction with visual area fits better with our predictions. And, if stimulus manipulations do modulate symmetry responses, but this pattern changes as a function of task, then we would witness a significant interaction between stimulus and task. Last, if stimulus manipulations modulate symmetry responses, in a different fashion across visual areas, but this pattern is only observed under a particular task, then we would expect to see a significant interaction between stimulus, visual area and task. On the whole, therefore, our predictions would most likely be supported by interaction terms.

For the coherence experiment, the three‐way ANOVA, including visual area, coherence, and task as factors. There were significant main effects of visual area *F*(13,78) = 11.31, *p* = 3.60 × 10^−13^, and symmetry coherence *F*(2,12) = 107.36, *p* = 2.20 × 10^−8^, but not of task *F*(1,6) = 3.43, *p* = .11. There were significant interactions between visual area and coherence *F*(26,156) = 13.42, *p* = 5.60 × 10^−28^, and visual area and task *F*(13,78) = 1.92, *p* = .04, but not coherence and task *F*(2,12) = 2.36, *p* = .14. There was no significant interaction between visual area, coherence, and task *F*(26,156) = 1.42, *p* = .10. This therefore confirmed that responses were modulated by symmetry and that those modulations differed across visual areas. While there were no significant effects of task or interaction between coherence and task, there was a significant interaction between visual area and task, indicating that symmetry responses were modulated differentially by visual area as a function of task. This modulation did not also differ as a function of coherence, although the three‐way interaction between coherence, visual area, and task was close to significance at *p* = .10.

While an omnibus test for all the experiments reported here is informative and matched many of our predictions, it is also important to examine our data with tests that are equally sensitive to effects as those used in studies that we are largely replicating (Sasaki et al., 2005). This measure is important to take as we do not want to inflate the risk of a type 2 error. We therefore used analyses to examine the relationship between brain responses and behavior to allow for direct comparisons with previous work (Sasaki et al., 2005). To detect visual areas whose responses scaled monotonically with coherence, much like behavior, we performed a linear trend analysis, marking visual areas that exhibit a significant linear trend with an asterisk on each graph. This analysis is identical to that used by Kohler et al. (2016), and very similar to that used by Sasaki et al. (2005), thus allowing us to detect behaviorally relevant neural responses with the same sensitivity as that achieved in studies we wish to emulate. During passive viewing, only TO2 was found to show a significant linear trend. During the symmetry task, however, V3 and all downstream areas with the exception of V7 exhibited a significant linear trend. These results demonstrate that when people are performing the symmetry task, a significant monotonic increase in neural symmetry responses is found that follows psychophysical responses to symmetry.

Results from the folds experiments are shown in Figure 4. As with the coherence data, we observed symmetry responses to emerge in V3 and continue through all downstream areas examined, with the strongest symmetry responses again found in VO1 and LOB. Similar to coherence, responses were also found to scale with the quality of symmetry in the stimulus, being weakest for onefold and strongest for fourfold patterns.

We again ran a three‐way ANOVA, including visual area, folds, and task as factors. There were significant main effects of visual area *F*(13,78) = 10.91, *p* = 8.33 × 10^−13^, symmetry folds *F*(2,12) = 66.52, *p* = 3.21 × 10^−7^, and task *F*(1,6) = 11.76, *p* = .01. There were significant interactions between visual area and symmetry folds *F*(26,156) = 8.82, *p* = 1.40 × 10^−19^, and visual area and task *F*(13,78) = 4.93, *p* = 0.03 × 10^−5^, but not symmetry folds and task *F*(2,12) = 0.41, *p* = .67. There was a significant interaction between visual area, symmetry folds, and task *F*(26,156) = 1.61, *p* = .04. The results therefore confirm the predictions we had; not all visual areas are symmetry selective, that selectivity scales with folds and that task enhances responses to symmetry in those areas exhibiting symmetry selectivity.

In contrast to the coherence experiments, responses appeared to scale reliably with the quality of the symmetry in the stimulus, namely, the increases in folds, irrespective of the task. That is, while responses were generally higher during the symmetry task as indicated by a main effect of task, similar scaling with increases in folds of symmetry were observed in the passive viewing and symmetry detection experiments, as indicated by the lack of interaction between task and folds. In line with our assessment of coherence, we test for linear trends of response with folds in each visual area for both tasks. For passive viewing, significant linear trends were found for V3 and all downstream areas examined, while for the symmetry detection task, significant linear trends were found for V3 and all downstream areas, except LO2. Visual areas that exhibit this significant linear trend are marked using an asterisk on each graph.

For the folds and coherence experiments, we obtained a stimulus localizer that allowed us to restrict analyses to the square 16° around fixation that was commensurate with the retinotopic locus of the stimulus. While this stimulus localizer produced consistent activation V1‐V4 (except in one participant where we were missing V4 in the left hemisphere), it produced little or no activation for areas downstream of V3/V4. We were therefore unable to utilize it for all of the retinotopically and functionally defined areas reported here. We did however check if the pattern of activation was similar between localizer‐masked and unmasked data. To this end, we correlated restricted, localizer data, with unrestricted, full‐field data. For the symmetry coherence experiment, unrestricted data correlated highly with localizer‐restricted data for both passive viewing *r* = .999, *p* = .001, *R* ^2^ = .998, and for symmetry detection, *r* = .996, *p* = .004, *R* ^2^ = .992. Overall, responses were slightly higher to the restricted data both for passive viewing (gradient of best fitting line = 1.071) and for symmetry detection (gradient of best fitting line = 1.164). For the symmetry folds experiment, unrestricted data correlated highly with localizer restricted data for both passive viewing *r* = .996, *p* = .004, *R* ^2^ = .992, and for symmetry detection *r* = .994, *p* = .006, *R* ^2^ = .988. As with the coherence experiment, responses were slightly higher to the restricted data both for passive viewing (gradient of best fitting line = 1.219) and for symmetry detection (gradient of best fitting line = 1.154). The high correlations reported here suggest that we would see the same pattern of results for unmasked full‐field data as we would for localizer‐restricted data. We therefore chose to report unrestricted data for all the experiments reported here.

### 3.2 Experiments comparing responses to frontoparallel and slanted symmetry

Results for the experiments comparing responses to frontoparallel and slanted symmetry are shown in Figure 5. The overall pattern of results by visual area was similar to the coherence and folds experiments discussed earlier, with symmetry responses emerging consistently in V3 and the largest symmetry responses observed in VO1 and LOB. Responses to the slanted plane were slightly lower than those to the frontoparallel plane, an effect that was more pronounced in the color detection task than in the symmetry detection task (compare panels C & B).

To explore the manipulations of slant, we ran a three‐way ANOVA, including visual area, slant, and task as factors. There were significant main effects of visual area *F*(13,78) = 9.07, *p* = 5.14 × 10^−11^, slant *F*(1,6) = 6.15, *p* = .048, but not of task *F*(1,6) = .45, *p* = .53. There was a significant interaction between visual area and task *F*(13,78) = 4.42, *p* = .15 × 10^−5^, but not visual area and slant *F*(13,78) = 1.50, *p* = .14, or slant and task *F*(1,6) = 1.25, *p* = .31. There was a significant interaction between visual area, slant, and task *F*(13,78) = 2.28, *p* = .01.

These results confirm the results of our earlier experiments, in that symmetry specific responses have an extrastriate origin as confirmed by the significant main effect of visual area. Here we extend those findings to show that introducing slant to the symmetry pattern produces a reduction in the observed responses, as evidenced by a main effect of slant. The effect of task produced a differential effect across visual areas as shown by the significant interaction between visual area and task. However, slant was not found to selectively differ by visual area or by task as shown by the absence of interactions in these cases. Importantly, however, the significant interaction between visual area, slant, and task demonstrates that slant led to a reduction in responses for certain visual areas, depending on the task performed.

To follow up on the main effect of visual area, we performed corrected one‐sample *t* tests for frontoparallel and slanted responses by each visual area, for the symmetry and color detection experiments. As in our earlier experiments isolated to the frontoparallel plane, we did not find any significant activity in V1 and V2. Activity was however found to emerge in V3 *p* < .01 to *p* < .05 and continue through the downstream areas examined, *p* < .001 to *p* < .05 indicating that the same visual areas were active for frontoparallel and slanted symmetry.

Because responses to onefold stimuli were the smallest (0.1–0.3% signal change) of all the stimuli we presented, and now these stimuli were additionally degraded by the introduction of slant, we would not expect it to be easy to find significant effects under these conditions. Owing to this lack of sensitivity, and more importantly, the fact we observed a significant three‐way interaction between visual area, slant, and task, we performed paired‐samples *t* tests (two‐tailed, uncorrected) by visual area for each experiment; cases where these *t* tests are significant are marked by asterisks on Figure 5. While for the symmetry detection experiment, only TO2 showed significantly lower responses to slanted symmetry, for the color detection experiment both V3 and VO1 showed significantly lower responses to slanted symmetry. These results are broadly consistent with Makin et al. (2015) who show a greater reduction to a symmetry‐related ERP component for a color discrimination task than for a symmetry discrimination task. It is also noteworthy that the significant reductions are found in V3 and VO1, the earliest and most strongly responding regions, respectively.

### 3.3 Correlations between experiments

**Figure 6:**
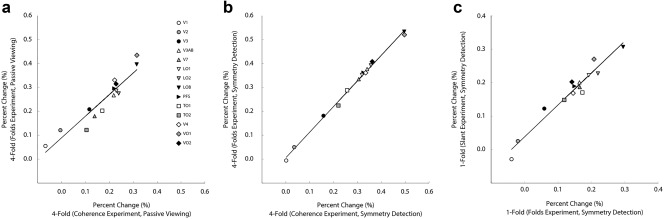
(a) Correlation between responses to fourfold stimuli in the coherence and folds experiments for passive viewing (same experimental session). (b) Correlation between responses to fourfold stimuli in the coherence and folds experiments for symmetry detection (same experimental session). (c) Correlation between responses to onefold, frontoparallel stimuli, in the folds and slanted symmetry experiments for symmetry detection (different experimental sessions)

One of the aims of our study was to understand how responses to symmetry are distributed across more contemporary definitions of retinotopic areas ‐ in other words, replicate the study by Sasaki et al. (2005). However, we also thought it important to examine the internal replicability of responses to symmetry which we obtained within our study. To check responses to symmetry were consistent across scans, we performed correlations between experiments in which the same stimuli were employed. Namely, for fourfold 100% coherence stimuli used in both the coherence and folds experiment, we performed correlations between responses to these stimuli for both the passive viewing and symmetry detection tasks, respectively. These correlations are from different scans performed in the same scan session (see methods) and are shown in Figure 6. Overall, it can be seen that responses to the same stimuli correlated highly between scans. For both passive viewing and symmetry detection, responses were found to correlate significantly *r* = .944, *p* = 4.02 × 10^−7^, *R* ^2^ = .891, and *r* = .998, *p* = 1.43 × 10^−15^, *R* ^2^ = 0.996, respectively. It appears that attending to the symmetry improved correlations over passive viewing as can be seen in the plots and the higher R‐squared value. These strong correlations suggest that participants' responses to stimuli were consistent within a scan session.

To check responses were consistent for data acquired across different sessions, performed on separate days, we performed a correlation between the onefold frontoparallel stimuli in the symmetry folds and slanted symmetry experiments for the symmetry detection condition (Figure 6c). This correlation was significant, *r* = .972, *p* = 6.65 × 10^−9^, *R* ^2^ = .945, suggesting that participants responses to stimuli were consistent across sessions performed on separate days.

## 4 DISCUSSION

Our results from the frontoparallel plane show symmetry‐specific responses to emerge in V3 and continue throughout visual cortex. Of the retinotopic areas examined, ventral occipital VO1 showed the strongest symmetry response which was similar in magnitude to responses observed in object selective cortex, LOB. Reponses generally increased with the quality of symmetry as both coherence and folds increased, consistent with recent work by other groups using EEG measures (Makin et al., 2016; Palumbo, Bertamini, & Makin, 2015). However, responses were only found to scale monotonically with increasing coherence during the symmetry detection task but not during passive viewing. While for folds of symmetry, this monotonic scaling was seen under both the passive viewing and the symmetry task. A key difference between these experiments is that the folds stimuli were always 100% coherence and thus showcased symmetry. During the coherence experiment, however, only one stimulus was presented at 100% coherence with the others degraded by varying degrees of noise. It is likely then, that under passive viewing, our response to the folds stimuli were driven by the perfect retinal regularity. This may explain why responses tended to scale monotonically with symmetry irrespective of the task in the folds experiment. Conversely, the reduction in retinal regularity caused by reduced coherence leads to a reduction in response when participants view passively compared to performing a symmetry task. It seems plausible, therefore, that top–down influences may explain why responses to symmetry degraded by noise are greater within the symmetry network during the symmetry task.

Similar to Sasaki et al. (2005), we observed symmetry‐related responses to begin in V3 and continue throughout visual cortex; we did not find symmetry‐specific activity in V1/V2. The general pattern of responses by visual areas is remarkably consistent between the data reported by Sasaki et al. (2005) and the data reported here. Sasaki et al. (2005) found the lowest symmetry response in V3 and the strongest in functionally localized LO. Their definition of LO shows overlap with our retinotopically defined VO1 and functionally localized LOB, the two areas where we also observe the largest symmetry‐related response. Sasaki et al. (2005) also identified V3A and V7, broadly overlapping with V3AB in this study, and V4d, broadly overlapping with LO1 and LO2 in this study. The responses Sasaki et al. (2005) observed in these areas were intermediate between those observed in V3 and LO. Similarly, we see responses in V3AB, LO1, and LO2 to sit in the mid‐range of responses, between those observed in V3 and VO1/LO. Last, Sasaki et al. (2005) identified MT+, corresponding to TO1 and TO2 in this study. The responses they observed in MT+ were small and similar to those observed in V3, echoing the symmetry responses we see in TO1 and TO2. While Sasaki et al. (2005) used a color task and found a reduction in symmetry‐related response compared to passive viewing, we found that having participants perform a symmetry‐related task increased symmetry‐related responses compared to passive viewing. Together these results suggest that when attention is directed toward symmetry, responses increase, but when attention is directed away from symmetry, such as during a color task, responses decrease. This task dependence has not emerged strongly from the EEG literature on symmetry (Höfel and Jacobsen, 2007a, 2007b; Jacobsen, & Höfel, 2003; Makin, Rampone, Pecchinenda, & Bertamini, 2013; Makin et al., 2014; Norcia et al., 2002; Rampone, Makin, & Bertamini, 2014; Wright, Makin, & Bertamini, 2017); however, perhaps because of an increased sensitivity of fMRI to attentional effects, we were able to detect such differences here.

To examine whether the same visual areas were recruited for processing symmetry in the slanted plane, we performed additional experiments utilizing onefold symmetrical patterns presented on both the frontoparallel and ± 50° planes. Onefold symmetry was used because it eliminates the perfect retinal symmetry found along the horizontal axis of two‐ and fourfold stimuli. We are confident, therefore, that our results are indicative of symmetry being extracted from the slanted plane as no residual frontoparallel information was available in our slanted stimuli. We found that responses to symmetry were generally similar across visual areas whether the stimuli were in the frontoparallel or slanted planes. However, subtle differences were found: (1) During the symmetry detection task, reductions in responses to slanted stimuli were small and only significantly lower than responses to frontoparallel symmetry in TO2 (Figure 5). (2) During the color task, however, reductions appeared larger and were significantly lower than responses to frontoparallel symmetry in V3 and VO1.

These results are broadly consistent with Makin et al. (2015) who found symmetry‐related ERP responses to frontoparallel and slanted symmetry to be similar during a symmetry discrimination task, but not during a color discrimination task, where no significant activation was seen for onefold slanted patterns. In contrast to Makin et al. (2015) however, we do not find neural responses to onefold slanted symmetry to be absent during a color detection task, only reduced compared to their onefold frontoparallel counterparts. This is perhaps unsurprising given the different methodologies and measurement techniques utilized across these two studies. Indeed, while during our color detection task, catch trails occurred infrequently (∼4% of trials), the study by Makin et al. (2015) had participants make color discrimination judgments on each trial. It is possible then, that the additional attentional requirements of these trial‐by‐trial color judgements led to a greater suppression of symmetry‐specific responses to slanted stimuli in the study by Makin et al. (2015). It is also of note that the measured EEG symmetry response is not always task dependent and can be similar across tasks, whether people are attending to regularity or some other feature such as the color of the dots (Höfel and Jacobsen, 2007a, 2007b; Jacobsen et al., 2003; Makin et al., 2014, 2013; Norcia et al., 2002; Rampone et al., 2014; Wright et al., 2017). Such task‐dependent differences may therefore only emerge for slanted stimuli.

Importantly, our results suggest that the same network of visual areas is called upon for computing both frontoparallel and slanted symmetry. Symmetry‐related activity was found to emerge in V3 and continue through all downstream areas examined independent of viewing angle. Furthermore, we observed LOB and VO1 to show the strongest symmetry response for symmetry on the slanted plane, echoing our findings for the frontoparallel plane. However, the greater reduction in responses to slanted compared with frontoparallel symmetry observed in the color detection experiment suggests that slanted symmetry may not be processed effectively under these conditions. It is possible that characteristics of feedforward processing are revealed during color detection. Specifically, drawing attention away from the orientation of the stimulus using a color task may interfere with the computation of slant in higher visual areas that likely feedback to visual areas processing symmetry. In this case, without a reliable estimate of slant, the putative normalization mechanism would be disrupted leaving neural responses driven primarily by the remaining regularities in the retinal image. Such an account would explain the reduction both we and Makin et al. (2015) have observed during a color task. Strict retinal regularity accounts of symmetry processing do not predict that the pattern of responses to symmetry in different planes should change when a different task is employed.

The retinal structure and normalization accounts of symmetry processing provide simple predictions for future studies looking to further investigate between these two putative mechanisms. If normalization does occur, then during engagement with symmetry we would expect to see greater feedback to e.g. V3 (for slanted compared to frontoparallel symmetry) from visual areas known to compute slant (Murphy, Ban, & Welchman, 2013; Tsutsui et al., 2001). However, if retinal structure is sufficient to extract symmetry from slanted planes then we would expect similar feedforward and feedback processes to be invoked independent of stimulus slant and task. To help answer these questions future investigations could draw from studies that have used transcranial magnetic stimulation (TMS) (Bona et al., 2014; Maus, Ward, Nijhawan, & Whitney, 2013; Wokke, Vandenbroucke, Scholte, & Lamme, 2012), and imaging of the laminar structure of visual cortex (Kok, Bains, van Mourik, Norris, & de Lange, 2016; Lawrence, Formisano, Muckli, & de Lange, 2017). Indeed, estimates of slant from disparity and texture cues have been found to be combined in V3B (Murphy et al., 2013), making this a candidate area for future TMS studies. While considering these future directions, it is important to note, after the suggestions of van der Vloed et al. (2005) that normalization may proceed in tandem with regularity discrimination. Indeed, Saunders and Knill (2001) have shown that symmetry can actually help retrieve the 3D orientation of surfaces, suggesting that normalization may be an integral part of the symmetry detection process, rather than necessarily having to precede it.

In Summary, the results of this study suggest that the symmetry‐specific responses emerge in V3 and continue throughout visual cortex, with the largest responses occurring in VO1 and LOB. While the same symmetry network appears to be involved in processing frontoparallel and slanted symmetry, our results suggest that attention to the stimulus is necessary for slanted symmetry to be processed most effectively. Specifically, our results indicate that normalization occurs naturally when attention is directed toward symmetry and orientation, but becomes interrupted when attention is directed away from these features. Future studies combining fMRI, TMS, and other techniques will be required to discern the exact processes underlying the recovery of symmetry from slanted planes in the human visual system.

## DECLARATION OF INTEREST

Conflicts of interest: none.

## References

[hbm24211-bib-0001] Amano, K. , Wandell, B. A. , & Dumoulin, S. O. (2009). Visual field maps, population receptive field sizes, and visual field coverage in the human MT+ complex. Journal of Neurophysiology, 102(5), 2704–2718. 19587323 10.1152/jn.00102.2009 PMC2777836

[hbm24211-bib-0002] Bertamini, M. , & Makin, A. D. J. (2014). Brain activity in response to visual symmetry. Symmetry (Basel), 6(4), 975–996.

[hbm24211-bib-0003] Bona, S. , Herbert, A. , Toneatto, C. , Silvanto, J. , & Cattaneo, Z. (2014). The causal role of the lateral occipital complex in visual mirror symmetry detection and grouping: An fMRI‐guided TMS study. Cortex, 51, 46–55. 24360359 10.1016/j.cortex.2013.11.004

[hbm24211-bib-0004] Brainard, D. H. (1997). The psychophysics toolbox. Spatial Vision, 10(4), 433–436. 9176952

[hbm24211-bib-0005] Brewer, A. A. , Liu, J. , Wade, A. R. , & Wandell, B. A. (2005). Visual field maps and stimulus selectivity in human ventral occipital cortex. Nature Neuroscience, 8(8), 1102–1109. 16025108 10.1038/nn1507

[hbm24211-bib-0006] Chen, C. C. , Kao, K. L. C. , & Tyler, C. W. (2007). Face configuration processing in the human brain: The role of symmetry. Cerebral Cortex (New York, N.Y. : 1991), 17(6), 1423–1432. 10.1093/cercor/bhl054 16923779

[hbm24211-bib-0007] DeYoe, E. A. , Carman, G. J. , Bandettini, P. , Glickman, S. , Wieser, J. , Cox, R. , … Neitz, J. (1996). Mapping striate and extrastriate visual areas in human cerebral cortex. Proceedings of the National Academy of Sciences of the United States of America, 93(6), 2382–2386. 8637882 10.1073/pnas.93.6.2382 PMC39805

[hbm24211-bib-0008] Dumoulin, S. O. , & Wandell, B. A. (2008). Population receptive field estimates in human visual cortex. NeuroImage, 39(2), 647–660. 17977024 10.1016/j.neuroimage.2007.09.034 PMC3073038

[hbm24211-bib-0009] Engel, S. A. , Glover, G. H. , & Wandell, B. A. (1997). Retinotopic organization in human visual cortex and the spatial precision of functional MRI. Cerebral Cortex (New York, N.Y. : 1991), 7(2), 181–192. 10.1093/cercor/7.2.181 9087826

[hbm24211-bib-0010] Grill‐Spector, K. , Kourtzi, Z. , & Kanwisher, N. (2001). The lateral occipital complex and its role in object recognition. Vision Research, 41(10–11), 1409–1422. 11322983 10.1016/s0042-6989(01)00073-6

[hbm24211-bib-0011] Grill‐Spector, K. , Kushnir, T. , Edelman, S. , Avidan, G. , Itzchak, Y. , & Malach, R. (1999). Differential processing of objects under various viewing conditions in the human lateral occipital complex. Neuron, 24(1), 187–203. 10677037 10.1016/s0896-6273(00)80832-6

[hbm24211-bib-0012] Höfel, L. , & Jacobsen, T. (2007a). Electrophysiological indices of processing symmetry and aesthetics. Journal of Psychophysiology, 21(1), 9–21. 10.1016/j.ijpsycho.2007.02.007 17400317

[hbm24211-bib-0013] Höfel, L. , & Jacobsen, T. (2007b). Electrophysiological indices of processing aesthetics: Spontaneous or intentional processes? International Journal of Psychophysiology, 65(1), 20–31. 17400317 10.1016/j.ijpsycho.2007.02.007

[hbm24211-bib-0014] Jacobsen, T. , & Höfel, L. (2003). Descriptive and evaluative judgment processes: Behavioral and electrophysiological indices of processing symmetry and aesthetics. Cognitive, Affective, & Behavioral Neuroscience, 3(4), 289–299. 10.3758/cabn.3.4.289 15040549

[hbm24211-bib-0015] Jenkins, B. (1983). Component processes in the perception of bilaterally symmetric dot textures. Perception &Amp; Psychophysics, 34(5), 433–440. 10.3758/bf03203058 6657447

[hbm24211-bib-0016] Jenkinson, M. , Bannister, P. , Brady, M. , & Smith, S. (2002). Improved optimization for the robust and accurate linear registration and motion correction of brain images. NeuroImage, 17(2), 825–841. 12377157 10.1016/s1053-8119(02)91132-8

[hbm24211-bib-0017] Kleiner, M. , Brainard, D. H. , Pelli, D. , Ingling, A. , Murray, R. , & Broussard, C. (2007). What's new in Psychtoolbox‐3? Perception, 36, 1–1.

[hbm24211-bib-0018] Kohler, P. J. , Clarke, A. , Yakovleva, A. , Liu, Y. , & Norcia, A. M. (2016). Representation of maximally regular textures in human visual cortex. Journal of Neuroscience, 36(3), 714–729. 26791203 10.1523/JNEUROSCI.2962-15.2016 PMC6602006

[hbm24211-bib-0019] Kok, P. , Bains, L. J. , van Mourik, T. , Norris, D. G. , & de Lange, F. P. (2016). Selective activation of the deep layers of the human primary visual cortex by top‐down feedback. Current Biology, 1–6. 10.1016/j.cub.2015.12.038 26832438

[hbm24211-bib-0020] Larsson, J. , & Heeger, D. J. (2006). Two retinotopic visual areas in human lateral occipital cortex. Journal of Neuroscience, 26(51), 13128–13115. 17182764 10.1523/JNEUROSCI.1657-06.2006 PMC1904390

[hbm24211-bib-0021] Lawrence, S. J. D. , Formisano, E. , Muckli, L. , & de Lange, F. P. (2017). Laminar fMRI: Applications for cognitive neuroscience. NeuroImage, 1–7. 10.1016/j.neuroimage.2017.07.004 28687519

[hbm24211-bib-0022] Locher, P. J. , & Smets, G. (1992). The influence of stimulus dimensionality and viewing orientation on detection of symmetry in dot patterns. Bulletin of the Psychonomic Society, 30(1), 43–46.

[hbm24211-bib-0023] Makin, A. D. J. , Rampone, G. , Wright, A. , Martinovic, J. , & Bertamini, M. (2014). Visual symmetry in objects and gaps. Journal of Vision, 14(3), 12–12. 10.1167/14.3.12 24610955

[hbm24211-bib-0024] Makin, A. D. J. , Wright, D. , Rampone, G. , Palumbo, L. , Guest, M. , Sheehan, R. , … Bertamini, M. (2016). An electrophysiological index of perceptual goodness. Cerebral Cortex (New York, N.Y. : 1991), 26(12), 4416–4434. 10.1093/cercor/bhw255 PMC5193141 27702812

[hbm24211-bib-0025] Makin, A. D. J. , Rampone, G. , & Bertamini, M. (2015). Conditions for view invariance in the neural response to visual symmetry. Psychophysiology, 52(4), 532–543. 25345662 10.1111/psyp.12365

[hbm24211-bib-0026] Makin, A. D. J. , Rampone, G. , Pecchinenda, A. , & Bertamini, M. (2013). Electrophysiological responses to visuospatial regularity. Psychophysiology, 50, 1045–1055. 23941638 10.1111/psyp.12082

[hbm24211-bib-0027] Malach, R. , Reppas, J. B. , Benson, R. R. , Kwong, K. K. , Jiang, H. , Kennedy, W. A. , … Tootell, R. B. (1995). Object‐related activity revealed by functional magnetic resonance imaging in human occipital cortex. Proceedings of the National Academy of Sciences of the United States of America, 92(18), 8135–8139. 7667258 10.1073/pnas.92.18.8135 PMC41110

[hbm24211-bib-0028] Maus, G. W. , Ward, J. , Nijhawan, R. , & Whitney, D. (2013). The perceived position of moving objects: Transcranial magnetic stimulation of area MT+ reduces the flash‐lag effect. Cerebral Cortex (New York, N.Y. : 1991), 23(1), 241–247. 10.1093/cercor/bhs021 PMC3513962 22302116

[hbm24211-bib-0029] Murphy, A. P. , Ban, H. , & Welchman, A. (2013). Integration of texture and disparity cues to surface slant in dorsal visual cortex. Journal of Neurophysiology, 110(1), 190–203. 23576705 10.1152/jn.01055.2012 PMC3727040

[hbm24211-bib-0030] Norcia, A. M. , Candy, T. R. , Pettet, M. W. , Vildavski, V. Y. , & Tyler, C. W. (2002). Temporal dynamics of the human response to symmetry. Journal of Vision, 2(2), 132–139. 12678588 10.1167/2.2.1

[hbm24211-bib-0031] Palumbo, L. , Bertamini, M. , & Makin, A. D. J. (2015). Scaling of the extrastriate neural response to symmetry. Vision Research, 117, 1–8. 26475086 10.1016/j.visres.2015.10.002

[hbm24211-bib-0032] Rampone, G. , Makin, A. D. J. , & Bertamini, M. (2014). Electrophysiological analysis of the affective congruence between pattern regularity and word valence. Neuropsychologia, 58, 107–117. 24746947 10.1016/j.neuropsychologia.2014.04.005

[hbm24211-bib-0034] Sasaki, Y. , Vanduffel, W. , Knutsen, T. , Tyler, C. , & Tootell, R. (2005). Symmetry activates extrastriate visual cortex in human and nonhuman primates. Proceedings of the National Academy of Sciences of the United States of America, 102(8), 3159–3163. 15710884 10.1073/pnas.0500319102 PMC549500

[hbm24211-bib-0035] Saunders, J. A. , & Knill, D. C. (2001). Perception of 3D surface orientation from skew symmetry. Vision Research, 41(24), 3163–3183. 11711141 10.1016/s0042-6989(01)00187-0

[hbm24211-bib-0036] Sayres, R. , & Grill‐Spector, K. (2008). Relating retinotopic and object‐selective responses in human lateral occipital cortex. Journal of Neurophysiology, 100(1), 249–267. 18463186 10.1152/jn.01383.2007 PMC2493478

[hbm24211-bib-0037] Szlyk, J. P. , Rock, I. , & Fisher, C. B. (1995). Level of processing in the perception of symmetrical forms viewed from different angles. Spatial Vision, 9(1), 139–150. 7626544 10.1163/156856895x00151

[hbm24211-bib-0038] Tootell, R. B. , Mendola, J. D. , Hadjikhani, N. K. , Ledden, P. J. , Liu, A. K. , Reppas, J. B. , … Dale, A. M. (1997). Functional analysis of V3A and related areas in human visual cortex. Journal of Neuroscience, 17(18), 7060–7078. 9278542 10.1523/JNEUROSCI.17-18-07060.1997 PMC6573277

[hbm24211-bib-0039] Tsutsui, K. , Jiang, M. , Yara, K. , Sakata, H. , & Taira, M. (2001). Integration of perspective and disparity cues in surface‐orientation‐selective neurons of area CIP. Journal of Neurophysiology, 86(6), 2856–2867. 11731542 10.1152/jn.2001.86.6.2856

[hbm24211-bib-0040] Tyler, C. W. , Baseler, H. A. , Kontsevich, L. L. , Likova, L. T. , Wade, A. R. , & Wandell, B. A. (2005). Predominantly extra‐retinotopic cortical response to pattern symmetry. NeuroImage, 24(2), 306–314. 15627573 10.1016/j.neuroimage.2004.09.018

[hbm24211-bib-0041] Vinberg, J. , & Grill‐Spector, K. (2008). Representation of shapes, edges, and surfaces across multiple cues in the human visual cortex. Journal of Neurophysiology, 99(3), 1380–1393. 18171705 10.1152/jn.01223.2007

[hbm24211-bib-0042] van der Vloed, G. , Csathó, Á. , & van der Helm, P. A. (2005). Symmetry and repetition in perspective. Acta Psychologica, 120(1), 74–92. 15932747 10.1016/j.actpsy.2005.03.006

[hbm24211-bib-0043] Wagemans, J. , van Gool, L. , & D'ydewalle, G. (1991). Detection of symmetry in tachistoscopically presented dot patterns: Effects of multiple axes and skewing. Perception &Amp; Psychophysics, 50(5), 413–427. 10.3758/bf03205058 1788030

[hbm24211-bib-0044] Wagemans, J. (1993). Skewed symmetry: A nonaccidental property used to perceive visual forms. Journal of Experimental Psychology: Human Perception and Performance, 19(2), 364., 8473845 10.1037//0096-1523.19.2.364

[hbm24211-bib-0045] Wagemans, J. , Van Gool, L. , Swinnen, V. , & Van Horebeek, J. (1993). Higher‐order structure in regularity detection. Vision Research, 33(8), 1067–1088. 8506646 10.1016/0042-6989(93)90241-n

[hbm24211-bib-0046] Wandell, B. A. , Brewer, A. A. , & Dougherty, R. F. (2005). Visual field map clusters in human cortex. Philosophical Transactions of the Royal Society of London. Series B, Biological Sciences, 360(1456), 693–707. 15937008 10.1098/rstb.2005.1628 PMC1569486

[hbm24211-bib-0047] Winawer, J. , Horiguchi, H. , Sayres, R. A. , Amano, K. , & Wandell, B. A. (2010). Mapping hV4 and ventral occipital cortex: The venous eclipse. Journal of Vision, 10(5), 1. 10.1167/10.5.1 PMC3033222 20616143

[hbm24211-bib-0048] Wokke, M. E. , Vandenbroucke, A. RE. , Scholte, H. S. , & Lamme, V. A F. (2012). Confuse your illusion: Feedback to early visual cortex contributes to perceptual completion. Psychological Science, 24, 63–71. 23228938 10.1177/0956797612449175

[hbm24211-bib-0049] Worsley, K. J. (2001). Statistical analysis of activation images. Global Governance.

[hbm24211-bib-0050] Wright, D. , Makin, A. D. J. , & Bertamini, M. (2017). Electrophysiological responses to symmetry presented in the left or in the right visual hemifield. Cortex, 86, 93–108. 27923173 10.1016/j.cortex.2016.11.001

